# Aggressive Paraneoplastic Seronegative Dermatomyositis Associated With Head and Neck Squamous Cell Carcinoma of Unknown Primary: A Case Report

**DOI:** 10.7759/cureus.115308

**Published:** 2026-08-27

**Authors:** Aicha Bourguiba, S M Shariar Islam, Arwa Fareah Ansar, Alaa AL Jarrad, Ishma Aijazi

**Affiliations:** 1 Department of Internal Medicine, Graduate Medical Education, Mohammed Bin Rashid University of Medicine and Health Sciences, Dubai Health, Dubai, ARE; 2 Department of Internal Medicine, College of Medicine, Mohammed Bin Rashid University of Medicine and Health Sciences, Dubai Health, Dubai, ARE; 3 Department of Internal Medicine, Dubai Hospital, Dubai Health, Dubai, ARE

**Keywords:** amyopathic dermatomyositis, head and neck cancer, leukocytoclastic vasculitis (lcv), paraneoplastic syndrome, squamous cell carcinoma of unknown primary

## Abstract

Dermatomyositis is a recognized paraneoplastic syndrome, with malignancy risk being an important consideration during evaluation. Its association with head and neck squamous cell carcinoma (SCC) is uncommon, and its occurrence with a presumed unknown primary has not been well documented.

We report the case of an 80-year-old man who presented with dysphagia, proximal weakness, and a characteristic rash. Serology was negative, but multiple malignancy alarm features (age, dysphagia, leukocytoclastic vasculitis on biopsy) prompted aggressive screening. Fluorodeoxyglucose (FDG)-PET/CT revealed a single hypermetabolic cervical lymph node; biopsy confirmed p16-positive SCC. No mucosal primary was identified despite laryngoscopy, and further biopsies could not be performed due to rapid functional decline. The patient proved refractory to immunosuppression and was unfit for oncologic therapy. He died three months after symptom onset.

This case illustrates the utility of clinical alarm features and early PET/CT in seronegative dermatomyositis, while highlighting how quickly the window for diagnosis and treatment can close.

## Introduction

Dermatomyositis is an idiopathic inflammatory myopathy characterized by proximal muscle weakness and distinctive cutaneous manifestations [1]. A well-recognized subset of cases occurs as a paraneoplastic phenomenon, with reported malignancy prevalence ranging from 15% to 30% [2,3]. Commonly associated malignancies include ovarian, lung, gastric, colorectal, and pancreatic carcinomas [4]. The absence of myositis-specific autoantibodies, older age at onset, dysphagia, and cutaneous leukocytoclastic vasculitis are established clinical predictors of underlying malignancy [5,6]. We report a case of paraneoplastic dermatomyositis in an 80-year-old man found to have human papillomavirus (HPV)-related squamous cell carcinoma of unknown primary (SCCUP) of the head and neck, a combination not previously described in the literature, to our knowledge.

## Case presentation

An 80-year-old man with a background of hypertension, type 2 diabetes mellitus, dyslipidemia, ischemic heart disease (post-coronary artery bypass grafting, 2019), and chronic kidney disease secondary to diabetes (baseline creatinine 1.0-2.0 mg/dL; estimated glomerular filtration rate 30-40 mL/min/1.73 m²) presented to the emergency department with a one-month history of progressive dysphagia, proximal limb weakness, and a worsening skin rash.

The rash was the first symptom to appear, coinciding temporally with his fifth subcutaneous injection of epoetin beta (4,000 units twice weekly), prescribed for anemia secondary to chronic kidney disease. He had received the same medication without incident over the preceding year. A dermatologist assessed him at that time and attributed the eruption to a drug reaction; epoetin beta was discontinued, and oral prednisolone was initiated. Despite this, the rash continued to worsen over the following weeks, prompting hospital admission.

Examination

**Figure 1:**
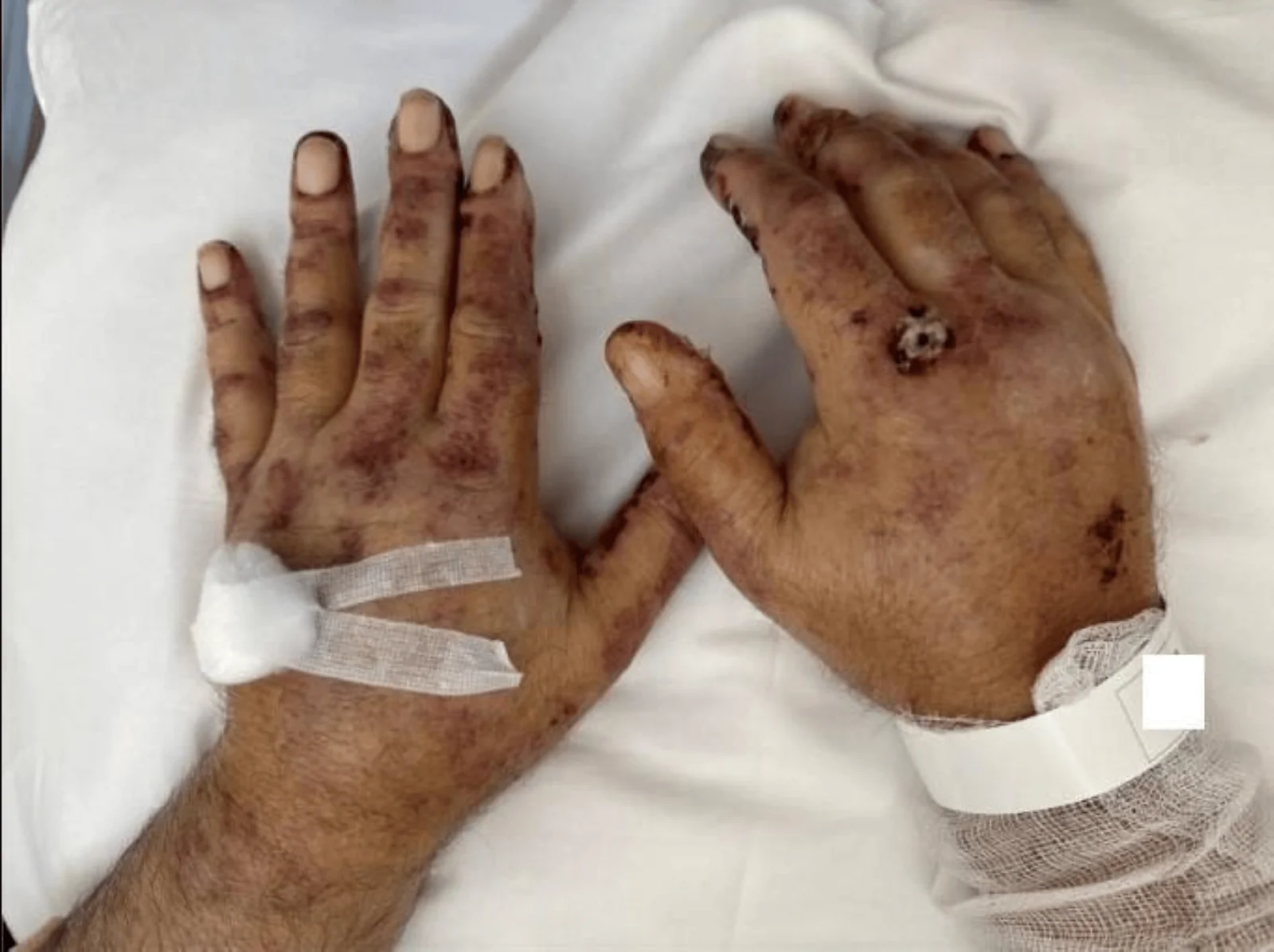
The patient's hands are showing digital nail fold infarcts and blackish discoloration of the fingertips, accompanied by a petechial and purpuric rash on the tips of the fingers.

On presentation, the patient was alert and oriented but visibly unwell. Facial and periorbital edema were prominent, with a violaceous erythema of the eyelids consistent with a heliotrope rash. Erythematous, scaly plaques were present over the dorsal hands (Figure 1) and forearms, and palpable purpuric lesions with hemorrhagic bullae were noted over the bilateral lower extremities, extending to the feet. Additional crusted lesions were present behind the ears, over the beard distribution, and in the periorbital region. Proximal muscle weakness was severe in both the upper and lower limbs, with muscle tenderness elicited on palpation. Speech was dysarthric with a nasal quality. The patient required assistance to mobilize.

Investigations

Laboratory investigations at the time of admission are presented in Table 1.

**Table 1:** Initial laboratory investigations at admission *CPK subsequently normalized after statin discontinuation. WBC, white blood cell count; ESR, erythrocyte sedimentation rate; PCT, procalcitonin; CPK (CK), creatine phosphokinase (creatine kinase); LDH, lactate dehydrogenase; INR, international normalized ratio; APTT, activated partial thromboplastin time; Ag, antigen; Ab, antibody.

Lab investigation	Result		Reference range	
WBC (x10^3^/uL)	9.9		3.6 - 11.0	
Hemoglobin (g/dL)	11.1		13.0 - 17.0	
Platelets (x10^3^/uL)	185		150 - 410	
C-reactive Protein (mg/l)	25.9		<5	
ESR (mm/hr)	39		0 - 20	
PCT (ng/ml)	0.12		<0.05	
Absolute reticulocyte count (x10^9^/L)	62.4		50 - 100	
Reticulocyte count (%)	2.21		0.5 - 2.5	
Haptoglobin (g/L)	0.98		0.30 - 2.00	
Infectious workup				
Hepatitis C Ab	Non-reactive		Non-reactive	
HIV 1 & 2 Ag & Ab	Non-reactive		Non-reactive	
Hepatitis B surface Ag	Non-reactive		Non-reactive	
Muscle Enzymes				
CPK (CK) (U/L)	1,658*		0 - 190	
LDH (U/L)	429		105 - 222	
Coagulation Profile				
Fibrinogen (mg/dl)	277		200 - 400	
Prothrombin time (seconds)	11.5		9.7 - 11.8	
INR	1.07		0.8 - 1.1	
APTT (seconds)	37		25.1 - 37.7	

Myositis-specific antibody testing and vasculitis workup were comprehensively negative (Table 2).

**Table 2:** Summary of secondary laboratory investigations CEA, carcinoembryonic antigen; PSA, prostate-specific antigen; CA 19-9, carbohydrate antigen 19-9; AFP, alpha-fetoprotein; CA 15-3, cancer antigen 15-3; ANA, antinuclear antibody; IFA, indirect immunofluorescence assay; anti-dsDNA, anti-double-stranded DNA; PR3, proteinase 3; MPO, myeloperoxidase; Mi-2, Mi-2 nuclear helicase antibody; ENA, extractable nuclear antigen; RNP/Sm, ribonucleoprotein/Smith; Sm, Smith; SS-A (Ro), Sjögren syndrome-related antigen A; SS-B (La), Sjögren syndrome-related antigen B; Scl-70, anti-topoisomerase I; Jo-1, histidyl-transfer RNA synthetase antibody.

Category	Lab investigation	Result		Reference range	
Tumor markers	CEA (ng/mL)	2.1		<3.8	
	PSA (total) (ng/mL)	0.11		<4	
	CA 19-9 (U/mL)	39.6		<39	
	AFP (IU/mL)	1.0		<5.8	
	CA 15-3 (U/mL)	10		<35.0	
Immunohematology	Direct Coombs test	Negative		Negative	
	Indirect Coombs test	Negative		Negative	
Autoimmune workup	Connective tissue workup	Negative		Negative	
	ANA by IFA	Positive 1/100		Negative	
	Anti-dsDNA	Negative		Negative	
	Lupus anticoagulant test	Negative		Negative	
	Cryoglobulin	Negative		Negative	
	Complement, C4 (g/L)	0.31		0.10 - 0.40	
	Complement, C3 (g/L)	0.98		0.90 - 1.80	
	PR3 antibody (CU)	<2.3		<20	
	MPO antibody (CU)	<3.2		<20	
	Mi-2 abs	Negative		Negative	
	ENA RNP/Sm	<0.1		<1.0	
	ENA Sm	<0.1		<0.1	
	ENA SS-A (RO)	<0.1		<0.1	
	ENA SS-B (LA)	<0.1		<0.1	
	ENA scleroderma SCL-70	<0.1		<0.1	
	ENA JO-1	<0.1		<0.1	
	ENA Centromeres	<0.1		<0.1	

Nerve conduction studies demonstrated a severe sensorimotor polyneuropathy, likely attributable in part to longstanding diabetes, though a vasculitic or paraneoplastic contribution could not be excluded. Electromyography confirmed severe myopathic changes in the proximal muscle groups, consistent with inflammatory muscle disease. MRI of the pelvis and thighs demonstrated signs of active myositis (Table 3). Flexible laryngoscopy identified cricopharyngeal and esophageal dysfunction, accounting for the patient's dysphagia.

**Table 3:** Imaging findings FDG, fluorodeoxyglucose; STIR, short tau inversion recovery.

Imaging study	Finding			
Chest radiograph	Mild congestive changes with cardiomegaly.			
FDG-PET/CT	Hypermetabolic left cervical lymph node and diffuse bilateral shoulder muscle uptake consistent with active myositis.			
Ultrasound neck	Left level III cervical lymph node (2.9 x 0.9 cm) with loss of fatty hilum and increased vascularity.			
MRI pelvis	Diffusely T2/STIR hyperintensity of pelvic and proximal thigh musculature consistent with active myositis.			

**Figure 2:**
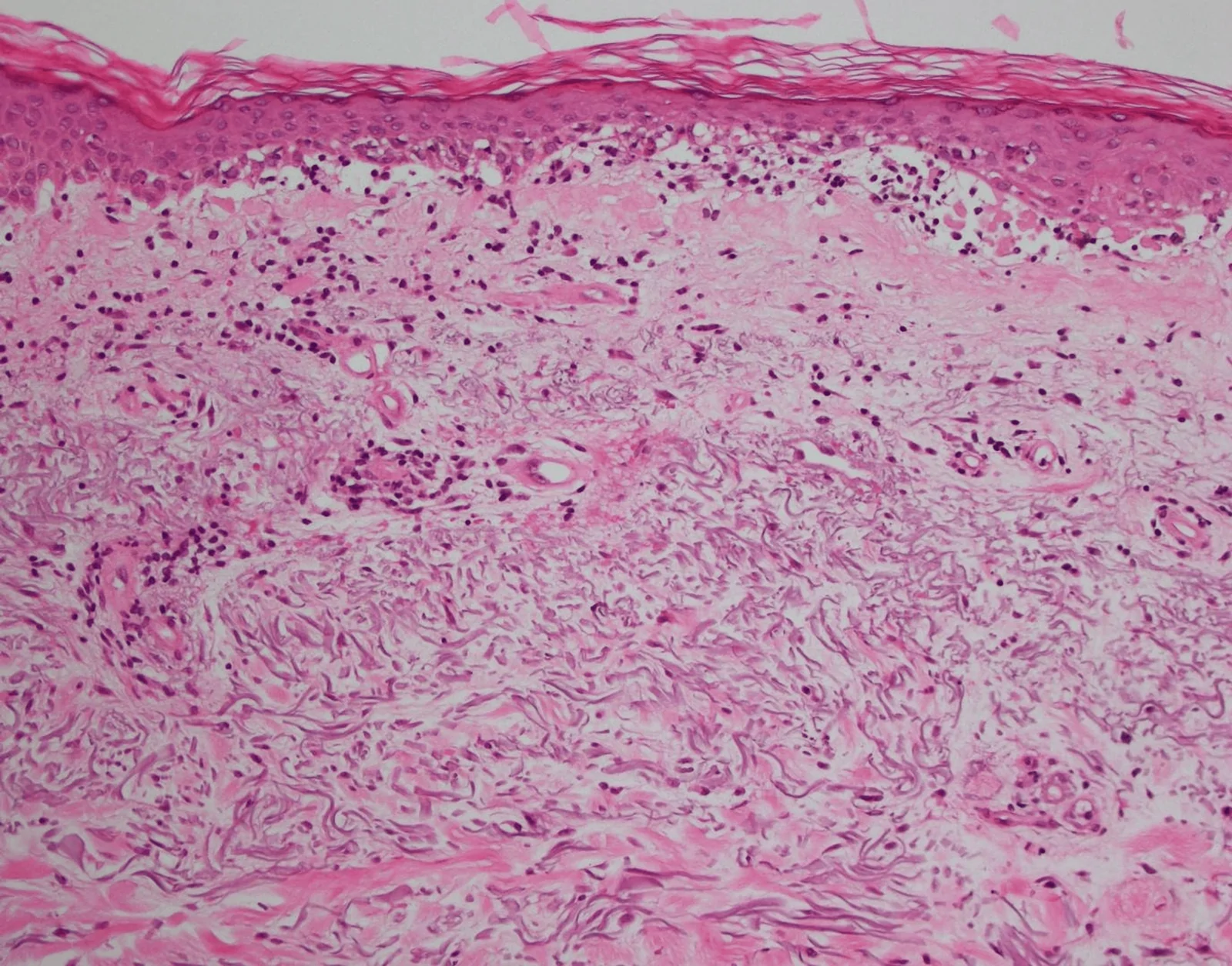
H&E-stained skin punch biopsy slide showing interface dermatitis, dermal myxoid degeneration, and solar elastosis.

Given that the cutaneous findings were not entirely classical for dermatomyositis, skin punch biopsies were taken from four separate sites. Histopathology (Table 4) demonstrated interface dermatitis (Figure 2) and florid leukocytoclastic vasculitis, with prominent involvement of medium and larger vessels in the deeper dermis. These findings were consistent with dermatomyositis-associated leukocytoclastic vasculitis.

**Table 4:** Histopathologic findings

Specimen	Finding			
Cervical lymph node biopsy	Squamous cell carcinoma, nonkeratinizing (G2, moderately differentiated)			
Immunohistochemical study on malignant cells	p16: Diffuse strong (block) positivity			
Initial skin punch biopsy (left hand)	Interface dermatitis			
Repeat skin biopsy	Florid leukocytoclastic vasculitis			

Malignancy workup

Given the patient's age, entirely negative autoantibody profile, dysphagia, and biopsy-confirmed leukocytoclastic vasculitis, all recognized alarm features for malignancy-associated dermatomyositis, an extensive malignancy screen was initiated. Tumor markers (Table 2), chest radiograph, CT of the chest, abdomen, and pelvis, upper gastrointestinal endoscopy, and colonoscopy were all unremarkable. CT of the paranasal sinuses showed no abnormality.

**Figure 3:**
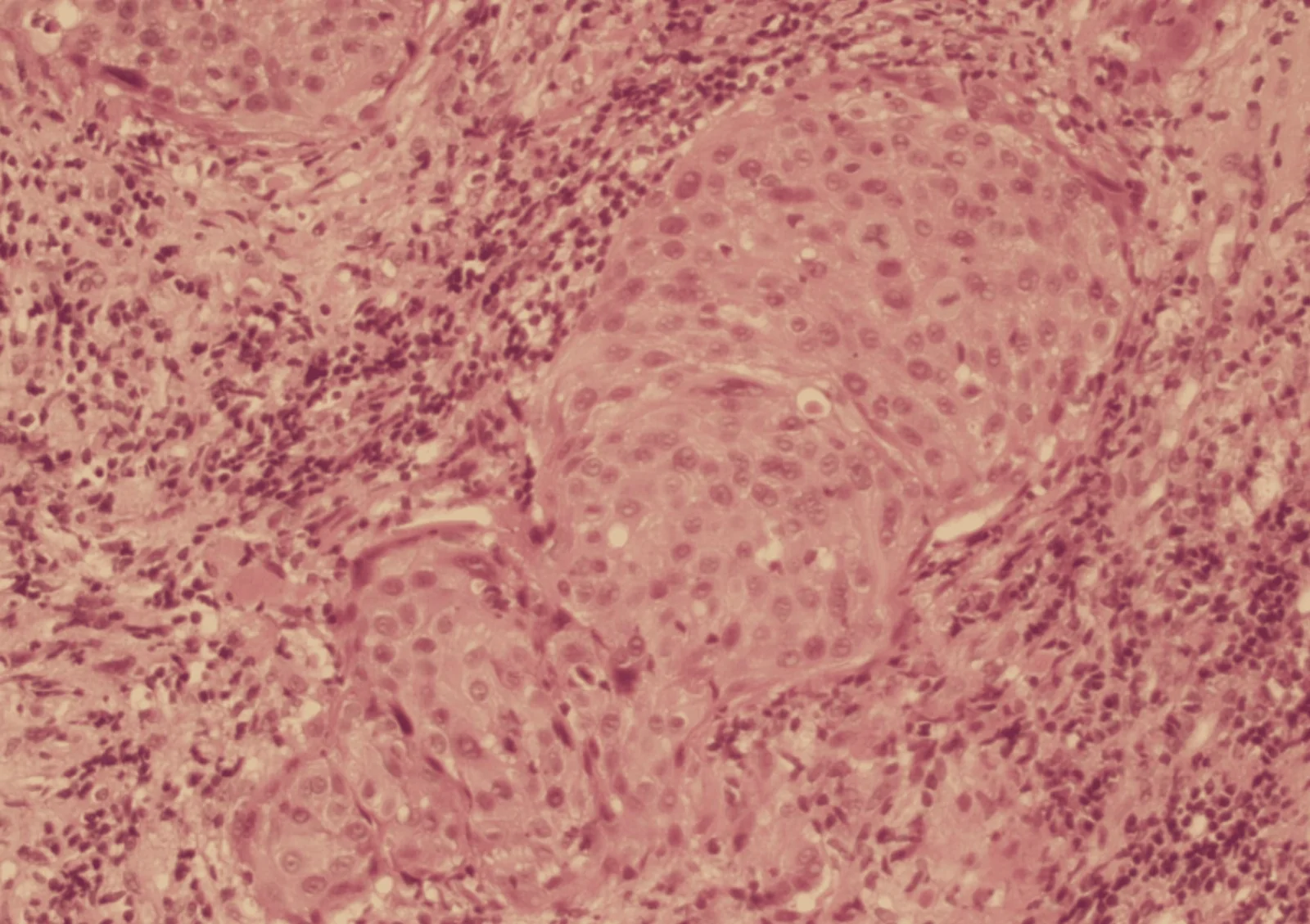
Cervical lymph node biopsy microscope slide showing a high-power view of an invasive tumour, showing nests of malignant squamous cells

**Figure 4:**
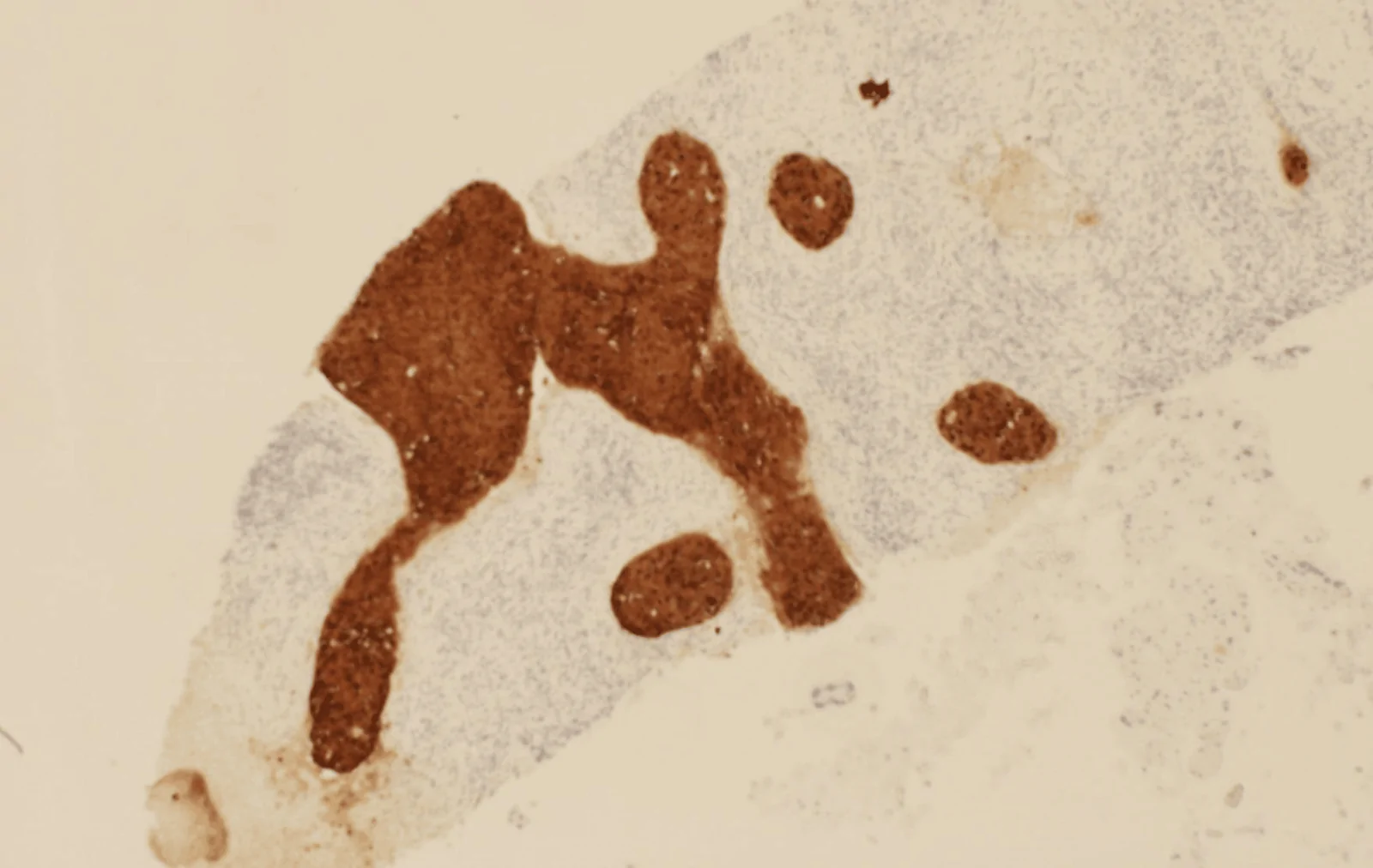
Cervical lymph node biopsy microscope slide showing tumor cells with diffuse strong p16 positive expression

Whole-body fluorodeoxyglucose (FDG)-PET/CT identified focally increased metabolic activity in a single left cervical lymph node at level IIA, alongside diffuse bilateral shoulder muscle uptake consistent with active myositis (Table 3). No other hypermetabolic lesions were identified. Biopsy of the involved lymph node revealed moderately differentiated, non-keratinizing squamous cell carcinoma (SCC, G2) (Figure 3). Immunohistochemistry for p16 was strongly positive, indicating probable HPV-related origin (Figure 4).

Repeat thorough laryngoscopic examination of the entire upper aerodigestive tract failed to identify a mucosal primary. Guided biopsies of the nasopharyngeal mucosa under general anesthesia were recommended by the ENT team, but declined by the patient's family, given the prohibitive perioperative risk in the context of his comorbidities and severely compromised functional status. A diagnosis of SCCUP of the head and neck was therefore established.

Treatment and clinical course

The patient was commenced on high-dose oral prednisolone at 1 mg/kg/day (80 mg daily), which produced modest and temporary relief of myositis-related symptoms and partial improvement of the cutaneous eruption. However, symptoms recurred promptly upon attempted dose reduction. The fingertip and toe lesions progressed to ulceration, necessitating regular wound care. Facial, periorbital, and limb edema worsened and proved refractory to diuresis and fluid restriction.

Oral intake remained severely compromised due to persistent cricopharyngeal dysfunction and associated swallowing muscle weakness, resulting in recurrent aspiration and chest infections. The patient subsequently developed respiratory difficulty requiring supplemental oxygen. High-resolution CT of the chest demonstrated bilateral infiltrates consistent with aspiration pneumonitis, with no evidence of interstitial lung disease. A percutaneous endoscopic gastrostomy tube was inserted to maintain adequate nutrition. A course of intravenous methylprednisolone pulse therapy and a subsequent course of intravenous immunoglobulin were both trialed without meaningful clinical response.

Oncological review concluded that the patient was not a suitable candidate for chemotherapy or radiotherapy, given his severely compromised functional status, multiple comorbidities, and no identifiable primary site.

During admission, the patient developed progressive pancytopenia (RBC 2.7x10^12^/L (4.5 - 5.5 x10^12^/L), WBC 2.1x10^9^/L (3.6 - 11 x 10^9^/L), platelets 29x10^9^/L (150 - 410 x10^9^/L)). Infectious causes, consumptive coagulopathy, and primary bone marrow pathology were systematically excluded; the bone marrow aspirate and peripheral blood film were both unremarkable. Supportive transfusions were administered, but the pancytopenia persisted without an identifiable cause.

The patient's condition deteriorated acutely with a sudden decline in mental status and unexplained respiratory distress, leading to cardiac arrest. He passed away approximately 60 days after admission and three months from the onset of his initial symptoms. Postmortem examination was declined by the family.

## Discussion

Dermatomyositis is a well-established paraneoplastic inflammatory myopathy in adults, with malignancy reported in approximately 15%-30% of cases across published cohorts, although estimates vary depending on study population and methodology [2,3]. The cancers most consistently implicated include ovarian, lung, gastric, colorectal, pancreatic, and breast carcinomas [4]. SCCUP is defined as any SCC for which the primary tumor cannot be identified despite appropriate evaluation [7]. SCCUP accounts for approximately 2%-5% of all head and neck cancers [8], while HPV p16 positivity has been reported in a substantial proportion of patients with SCCUP (overall median prevalence of 86.2%) [9]. Paraneoplastic dermatomyositis associated with head and neck SCCUP is exceptionally rare. Kim et al. previously described paraneoplastic dermatomyositis associated with SCCUP presenting as a pelvic and para-aortic mass [10]. Conversely, head and neck p16-positive cases triggering dermatomyositis, such as the tonsillar case reported by Vogel et al. [11], have featured an identifiable mucosal primary. Our case is distinctive in presenting with florid dermatomyositis associated with p16-positive cervical nodal SCC without an identifiable mucosal primary.

The diagnostic process in our patient illustrates the importance of maintaining suspicion for malignancy despite negative serological testing when clinical risk factors are present [5,12]. The myositis-specific antibody panel was entirely negative, including anti-Jo-1, anti-Mi-2, anti-MDA5, anti-RNP, anti-Ro, and anti-La. Three additional alarm features were present, namely age greater than 45 years, dysphagia, and biopsy-confirmed cutaneous leukocytoclastic vasculitis. Hunger et al. demonstrated that leukocytoclastic vasculitis on skin biopsy carries significant predictive value for underlying malignancy in dermatomyositis [6]. The convergence of these clinical and histopathological features therefore prompted further evaluation for an occult malignancy despite the negative antibody panel.

Initial malignancy evaluation, including tumor markers, CT of the chest, abdomen, and pelvis, upper gastrointestinal endoscopy, and colonoscopy, was unrevealing. Given the persistent clinical suspicion, whole-body FDG-PET/CT was subsequently performed and identified a single hypermetabolic left cervical lymph node. Biopsy demonstrated moderately differentiated, non-keratinizing SCC with strong p16 positivity, consistent with HPV-related origin. Biopsy of the skin lesions concurrently demonstrated leukocytoclastic vasculitis. These findings supported a diagnosis of paraneoplastic dermatomyositis associated with HPV-related SCC presenting in a cervical lymph node.

The subsequent diagnostic evaluation must be considered within the established framework for SCCUP. The standard workup includes comprehensive head and neck examination, flexible laryngoscopy, cervical lymph node biopsy, and imaging with FDG-PET/CT to identify an occult primary site. When the primary remains unidentified, examination under anesthesia with panendoscopy, directed biopsies of suspicious areas, and tonsillectomy are generally recommended, as the palatine and lingual tonsils represent common sites of occult primary tumors [8,13]. In our patient, extensive clinical and endoscopic evaluation failed to identify a mucosal primary. Further examination under anesthesia, including directed nasopharyngeal biopsies and tonsillectomy, was not performed after the family declined these procedures in light of the perioperative risks. Accordingly, the malignancy is best described as a presumed SCCUP.

The rarity of this presentation is further supported by the existing literature. A 2026 systematic review of paraneoplastic cutaneous syndromes in oral and oropharyngeal SCC identified dermatomyositis as the second most common entity among 46 reported cases, with dermatomyositis preceding the cancer diagnosis in all nine cases in which this relationship was documented. HPV status was reported in only five cases, and no cases originated from the Middle East [14]. The closest published case report by Vogel et al. described paraneoplastic dermatomyositis as the presenting feature of p16-positive SCC of the left tonsil [11]. There, the primary tumor was identified through standard evaluation, allowing definitive chemoradiation. In contrast, our patient's primary site remained unidentified, limiting the available oncologic treatment pathway.

Our patient's clinical course was characterized by rapidly progressive and treatment-refractory dermatomyositis despite high-dose corticosteroids and intravenous immunoglobulin, resulting in profound functional decline. Paraneoplastic dermatomyositis may precede identification of an associated malignancy by several months, and the risk of malignancy is particularly elevated during the first three years following dermatomyositis diagnosis [15]. In this case, however, the rapid deterioration substantially narrowed the window for both further diagnostic evaluation and cancer-directed treatment. HPV-related SCCUP of the head and neck generally carries a favorable prognosis, with reported three-year survival rates of up to 74% [13]. The death of our patient within three months of symptom onset was therefore markedly discordant with these outcomes. The poor prognosis in this case may have been driven less by tumor burden than by the severity and rapid progression of the paraneoplastic syndrome.

This case highlights an important practical consideration in patients with seronegative dermatomyositis and multiple malignancy-associated alarm features: the timing of malignancy screening may be as important as the screening modality itself. Current guidance recommends malignancy screening in dermatomyositis patients with recognized risk features but does not specify a time-sensitive threshold beyond which functional decline may foreclose further investigation or treatment [5,16]. Our case suggests that this window may close faster than is generally appreciated, particularly in older patients presenting with dysphagia and vasculitic skin involvement. Earlier escalation to comprehensive malignancy screening, including FDG-PET/CT when clinically appropriate, may therefore be warranted in this high-risk subgroup before significant functional reserve is lost.

## Conclusions

This case illustrates paraneoplastic dermatomyositis associated with presumed HPV-related SCCUP of the head and neck, diagnosed based on the most extensive workup performed. The diagnosis was reached through recognition of established alarm features in a seronegative patient, with FDG-PET/CT proving pivotal when conventional investigations were unrevealing. Paraneoplastic dermatomyositis can predate the onset of malignancy, treatment options can be limited, and dermatomyositis associated with malignancy might show minimal response to high-dose intravenous steroids. Although SCC associated with HPV is slow-growing and carries a favorable prognosis, aggressive paraneoplastic dermatomyositis can be life-threatening and refractory to steroids and immunosuppressants. 
